# Daytime Napping and Nighttime Sleep Duration with Incident Diabetes Mellitus: A Cohort Study in Chinese Older Adults

**DOI:** 10.3390/ijerph18095012

**Published:** 2021-05-09

**Authors:** Li Lin, Ciyong Lu, Weiqing Chen, Vivian Yawei Guo

**Affiliations:** Department of Epidemiology, School of Public Health, Sun Yat-Sen University, Guangzhou 510080, China; linli58@mail2.sysu.edu.cn (L.L.); luciyong@mail.sysu.edu.cn (C.L.); chenwq@mail.sysu.edu.cn (W.C.)

**Keywords:** daytime napping, nighttime sleep duration, diabetes mellitus, Chinese elderly

## Abstract

Background: We aimed to examine the longitudinal associations between daytime napping and nighttime sleep duration with the risk of diabetes mellitus (DM) among Chinese elderly using data from the China Health and Retirement Longitudinal Study (CHARLS). Methods: A cohort study was conducted among 2620 participants aged 60 years or above. Information on daytime napping and nighttime sleep duration was self-reported during the 2011 baseline survey. DM status during the 2015 follow-up survey was confirmed according to the American Diabetes Association criteria. Results: Individuals with long daytime napping (>1 h/day) had increased risk of developing DM than non-nappers (adjusted RR = 1.52, 95%CI: 1.10, 2.10). In addition, we observed a U-shaped association between nighttime sleep duration and incident DM risk. We further found that nappers with <4 h of nighttime sleep, and those with >1 h of daytime napping and >6 h nighttime sleep had approximately two-fold elevated risk of DM, compared to non-nappers with 6–8 h of nighttime sleep. Conclusion: Long daytime napping and extreme nighttime sleep duration were associated with increased DM risk among Chinese elderly. There was a joint effect of long daytime napping and nighttime sleep duration on the risk of DM.

## 1. Introduction

Diabetes mellitus (DM) is one of the major public health concerns globally. It is estimated that more than 460 million people worldwide are living with DM in 2019, which accounted for 9.3% of the adults older than 20 years [1]. As the country that has the largest number of people with DM, the latest national survey indicated that around 12.8% of the adults in China were suffering from DM [2]. This figure was even higher among Chinese older people, with an overall prevalence of 28.8% in people aged 60–69 years and 31.8% in people aged 70 years and above [2], indicating more attention should be paid to this vulnerable groups in order to reduce their risk of DM and its accompanying complications and mortality [3,4].

An optimal sleep duration is essential for maintaining the normal function in cognitive, physical and psychological process. A previous meta-analysis has confirmed a U-shaped association between sleep duration and DM risk, suggesting that both short and long sleep durations could increase the risk of DM [5]. In recent years, several studies have shown that daytime napping, a prevalent practice among the elderly, especially for people living in Mediterranean countries or China, was also associated with the risk of DM [6,7,8,9,10]. For example, a prospective study of 174,542 participants aged between 50 to 71 years in the US suggested that people with daytime napping had increased risk of DM compared to non-nappers [6]. A similar detrimental effect of daytime napping on DM risk was also demonstrated in a British study [9]. However, other studies did not find consistent associations between the two [11,12,13]. A study even showed that short daytime napping (<1 h per day) could reduce the risk of developing DM [12]. We previously conducted a meta-analysis and found that individuals with long daytime napping over 1 h per day seemed to be associated with increased risk of incident DM, but not for those who slept less than 1 h per day [7]. However, significant heterogeneity indicated the need for further studies [7].

Recently, a cross-sectional study among Chinese population from the China Health and Retirement Longitudinal Study (CHARLS) further demonstrated the detrimental effect of long daytime napping (>90 min per day) on prediabetes and DM [8]. However, the longitudinal associations in elderly from the same study was not clear. Therefore, in this cohort study, we aimed to evaluate the associations between daytime napping and nighttime sleep duration with incident DM risk among older Chinese population (>60 years) using data from CHARLS. We further assessed the joint effects of daytime napping and nighttime sleep on the risk of developing DM.

## 2. Methods

### 2.1. Study Design and Population

**Figure 1:**
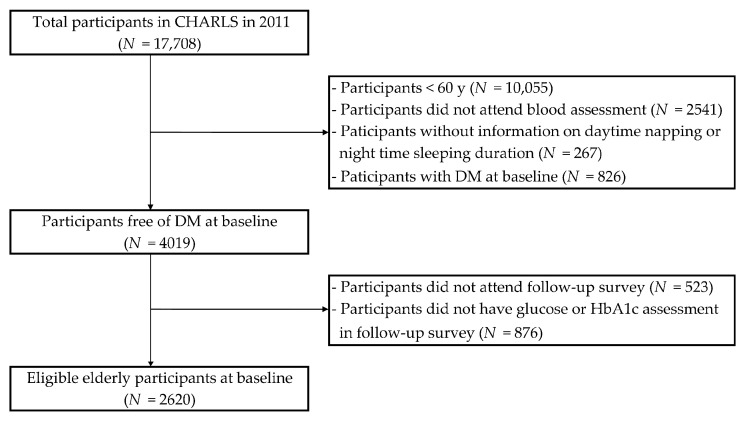
Study flowchart of participant selection.

This cohort study used data from CHARLS, a nationally representative longitudinal study of respondents aged 45 years and older [14]. The detailed description of CHARLS has been documented elsewhere [14]. Briefly, the baseline survey was conducted in 2011 and a total of 17,708 individuals were randomly selected from 450 villages/resident communities of 28 provinces in China with a probability-proportional-to-size sampling strategy. The follow-up survey was conducted every two years among previous respondents, with a small share of new participants recruited each time. After excluding 10,055 participants aged <60 years, 2541 without blood assessment of glucose or glycated hemoglobin (HbA1c), 267 without information on daytime napping or nighttime sleeping duration, and 826 with DM at baseline, 4019 participants free of DM remained in the baseline survey (Figure 1). We further excluded 523 individuals who did not attend the 2015 survey and 876 individuals without blood assessment of glucose or HbA1c during the follow-up survey. Finally, a total of 2620 eligible elderly were included in the analysis. The current study follows the Strengthening the Reporting of Observational Studies in Epidemiology (STROBE) reporting guideline for cohort studies (Supplementary Table S1).

### 2.2. Ascertainment of DM

Each participant was asked about whether he/she has been diagnosed with DM by a physician previously and whether they were on any glucose lowering drugs or insulin treatment at the time of the survey. In addition, venous blood was collected from each participant with their fasting status recorded. The blood samples were first kept in local hospitals, then transported to Peking University in Beijing and stored at −80 °C until measurement. Plasma glucose concentration were measured using hexokinase method, of which the inter-assay coefficient of variation was <0.40%. HbA1c levels were assessed with high-performance liquid chromatography method and the inter-assay coefficient of variation was <1.16% [15]. According to the American Diabetes Association criteria, DM was confirmed if any of the following criteria was met: (1) fasting plasma glucose ≥ 7.0 mmol/L; (2) random plasma glucose ≥ 11.1 mmol/L; (3) HbA1c ≥ 6.5%; (4) self-reported DM diagnosed by a physician; and (5) on anti-diabetic drugs or insulin treatment [16].

### 2.3. Daytime Napping and Nighttime Sleep Duration

Participants were asked about the duration of nap after lunch and the actual night sleep duration in the past month. Daytime napping was further classified into three groups: none, ≤1 h, and >1 h per day. Nighttime sleep duration was categorized into four groups: ≤4 h, 4–6 h, 6–8 h, and >8 h per day.

### 2.4. Covariates

Information on socio-demographic and lifestyle variables including age, gender, educational attainment (below primary school, primary school, and middle school or above), race (Han ethnicity and other minorities), area of residence (urban and rural), marital status (not married, and married or cohabitated), smoking status (never and ever smoker), and drinking status (never and ever drinker) were collected through face-to-face interviews from each participant.

The standing height was measured by a standardized stadiometer (Seca™213, Seca Co., Ltd., Hangzhou, China) and the weight was evaluated by a validated scale (Omron™ HN-286 scale, Krill Technology Co., Ltd., Yangzhou, China). BMI was calculated as weight (kg) divided by the square of height (m^2^). Based on the recommendation for Chinese adults, obesity was defined as BMI ≥ 28 kg/m^2^ [17]. Waist circumference (WC) was measured using a soft tape to the nearest 0.1 cm and central obesity was defined with a WC ≥90 cm for males and ≥85 cm for females [18]. Systolic blood pressure (BP) and diastolic BP were recorded three times with at least 45 s intervals by a digital sphygmomanometer (Omron™ HEM-7200, Dalian, China). Total cholesterol, high-density lipoprotein cholesterol (HDL-c), low-density lipoprotein cholesterol (LDL-c) and triglycerides were measured by enzymatic colormetric test. 

### 2.5. Statistical Analysis

Description of the baseline characteristics was presented as mean and standard deviation (SD) for continuous variables and frequency with percentages for categorical variables. Differences across daytime napping groups or nighttime sleep duration groups were compared by analysis of variance (ANOVA) for continuous variables and Chi-square tests for categorical variables.

Logistic regression analyses were applied to evaluate the associations of daytime napping and nighttime sleep duration with DM. In the adjusted Model 1, age, gender, race, education level, area of residence, marital status, drinking and smoking status, as well as systolic BP were included. Model 2 additionally adjusted for nighttime sleep duration (when the independent variable was daytime napping), and daytime napping (when the independent variable was nighttime sleep duration). To account for the mediating role of BMI and total cholesterol level, Model 3 further controlled for these two variables. Relative risks (RRs) with 95% confidence intervals (CI) were reported. We further evaluated the combined effects of daytime napping and nighttime sleep duration on the risk DM using the same covariates adjusted in Model 1. Non-nappers who slept for 6–8 h per night were set as the reference group in the analysis. Multivariable generalized additive model was also constructed to explore the curvilinear relationship between nighttime sleep duration and risk of incident DM, with value of effective degree of freedom (EDF) reported, which refers to the degree of curvature for smooth terms. 

All analyses were performed with Stata statistical software Version 15.0 (StataCorp LLC, Bryan, TX, USA). Two-tailed *p*-value < 0.05 indicated statistical significance.

## 3. Results

Among the 2620 elderly free of DM at baseline, 51.0% were males and the mean age was 66.9 ± 5.8 years. Nearly half of the participants (47.1%) reported no daytime napping and 18.4% had long daytime napping over 1 h per day. Comparison of baseline characteristics according to different groups of daytime napping duration is presented in Table 1. In general, participants with >1 h of daytime napping were more likely to be males, ever smokers and drinkers. The mean sleep duration was the longest among participants who had long daytime napping (6.7 ± 1.9 h) and the shortest among non-nappers (5.9 ± 2.1 h). During the four-year of follow-up, 358 (13.7%) participants developed DM. Although the differences of DM incidence across the three daytime napping groups were not statistically different, we observed a significant increasing trend (12.6% in non-nappers; 13.6% in participants with <1 h of daytime napping; and 16.6% in participants with >1 h of daytime napping, *p* = 0.091 for differences and *p* = 0.037 for trend test).

Table 2 shows the characteristics across different groups by nighttime sleep duration. Approximately 36.5% of participants reported an optimal 6–8 h of nighttime sleep, while 20.8% and 8.5% of the individuals had ≤4 h and >8 h of sleep at night, respectively. Compared to those with 6–8 h of nighttime sleep, participants with ≤4 h of nighttime sleep were more likely to be females, less educated, urban residents, currently not married, and were less likely to be ever smokers or ever drinkers. Additionally, those with >8 h of nighttime sleep were less educated, and more likely to be an urban resident and with other ethnicities, compared to the reference group. The mean daytime napping duration gradually increased with the nighttime sleep duration, with 0.49 (SD: 0.03) hours in those slept ≤4 h at night and 0.79 (SD: 0.06) hours in participants with >8 h of nighttime sleep per day.

The associations between daytime napping and nighttime sleep duration with the risk of DM are presented in Table 3. Compared with non-nappers, those with >1 h of daytime napping had 39% (95%CI: 3–86%) increased risk of developing DM in the crude model. With adjustment for covariates, the significant association was still present (RR: 1.60, 95%CI: 1.17–2.19 in the adjusted Model 1; RR: 1.63, 95%CI: 1.18–2.24 in the adjusted Model 2), while the effect estimate was somewhat attenuated after introducing the mediators of BMI and total cholesterol in adjusted model 3 (RR: 1.52, 95%CI: 1.10–2.10). However, shorter daytime napping (≤1 h per day) seemed not to be a significant risk factor to DM in both crude and adjusted models. In terms of the association between nighttime sleep duration and risk of DM, no significant association was found in the crude model. Nevertheless, with controlling for confounders in adjusted Model 1 and Model 2, participants who slept for >8 h during night were at higher risk of developing DM compared to those with the optimal 6–8 h of nighttime sleep. The associations remained significant even after further including the mediators of BMI and total cholesterol level in adjusted Model 3 (RR: 1.55, 95%CI: 1.01–2.38). Additionally, we observed significant association between short nighttime sleep duration and incident DM after inclusion of BMI and total cholesterol into the model (RR: 1.45, 95%CI: 1.04–2.01 in adjusted Model 3). In contrast, individuals with 4–6 h of nighttime sleep per day was not significantly associated with the risk of DM compared to the reference group in both crude and adjusted models. We further observed a non-linear association between nighttime sleep duration and incident DM risk (EDF = 1.951 > 1), and the lowest risk was observed in participants with nighttime sleep around 7 h (Supplementary Table S2). Supplementary Figure S1 shows an approximately smoothing “U-shape” of such association.

**Figure 2:**
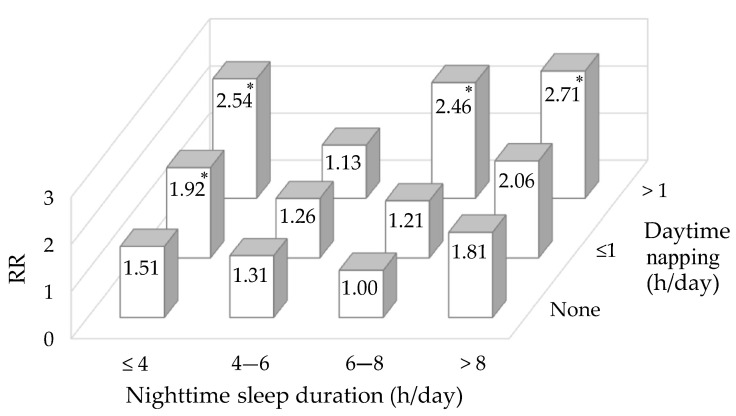
Combined effects of daytime napping and nighttime sleep duration on the risk of diabetes mellitus. * *p* < 0.05. Adjusted for age, gender, race, education level, area of residence, marital status, drinking and smoking status and systolic BP. Abbreviation: RR: relative risk.

We further evaluated the combined effects of daytime napping and nighttime sleep duration on the risk of DM (Figure 2). In each daytime napping group, there was a U-shaped association between nighttime sleep duration and risk of DM. Compared to non-nappers with 6–8 h of nighttime sleep, nappers who slept ≤4 h at night per day had over two-fold increased risk of developing DM (RR: 1.92, 95%CI: 1.10–3.34 for those with ≤1 h of napping; RR: 2.54, 95%CI: 1.21–5.30 for those with >1 h of napping). Participants with long daytime napping (>1 h) and nighttime sleep duration over 6 h also showed higher DM risk (RR: 2.46, 95%CI: 1.49–4.07 for those with 6–8 h of nighttime sleep; RR: 2.71, 95%CI: 1.26–5.82 for those with >8 h of nighttime sleep).

## 4. Discussion

After four years of follow-up, we found that long daytime napping (>1 h), as well as long (>8 h) and short (≤4 h) nighttime sleep duration was positively associated with the risk of developing DM. More importantly, nappers with short nighttime sleep duration (<4 h) as well as participants with >1 h of daytime napping and >6 h of sleep per night had more than two times increased risk of incident DM than non-nappers with 6–8 h of nighttime sleep. Such associations remained significant after adjustment for BMI and total cholesterol level.

The present study indicated that long daytime napping (>1 h) was associated with increased DM risk in Chinese elderly, which was in line with previous studies [19,20]. However, inconsistent findings were also reported [8,9,21,22]. For example, a prospective study among middle-aged and older adults found that daytime nappers were at higher risk of DM irrespective of their nap duration [9], while some other studies indicated an increased DM risk only among those with >90 min of daytime napping [8,22]. Additionally, a study conducted in the US showed non-significant association between daytime napping and DM among the elderly [21]. Such discrepancies might be explained by the different populations across studies. A previous study suggested that although the risk of DM risk increased among frequent daytime nappers, the magnitude of the associations was different by races [23], which could partly explain the heterogeneity between our study and those conducted in other populations with different genetic background. In addition, some studies further performed analyses among those who had a nap for >90 min during the day [8,12,24]. In our study population, there were only 89 individuals who reported a daytime napping between 61–90 min. In addition, our previous meta-analysis showed that daytime napping over 1 h per day was already associated with increased risk of DM [7]. Therefore, we combined participants with >1 h daytime napping together in the analysis. 

The mechanism underlying the association between long daytime napping and increased risk of DM is not clearly understood. A number of different pathways might explain the observed associations. First, awakening from long daytime napping could amplify activity of the sympathetic nervous system, leading to disruption of the sympatho-vagal balance and activation of the renin-angiotensin system [25,26]. Such changes could subsequently inhibit the function of pancreatic beta-cell, thus resulting in decreased insulin secretion and impaired glucose regulation [25,26]. Second, long daytime napping could cause elevation in post-nap cortisol level, which might induce insulin resistance and abnormal glucose metabolism [27]. Third, it is suggested that long daytime napping could result in imbalanced circadian rhythms [28], which was associated with defective beta-cell function, hypoinsulinaemia, impaired glucose tolerance and ultimately the development of DM [29,30]. Fourth, individuals who had long nap during the day were more likely to suffer from obstructive sleep apnea (OSA) [31], which could cause intermittent hypoxia and sleep fragmentation, and subsequently increase the sympathetic activation and nocturnal cortisol level [32], thus leading to insulin resistance and glucose intolerance, as well as DM [33,34]. Fifth, long daytime napping could elevate the levels of pro-inflammatory markers, such as interleukin-6 (IL-6), C-reactive protein and fibrinogen [35,36], posing a threat to increased risk of DM [37]. Last but not least, evidence has shown that long daytime nappers had lower leptin level and elevated ghrelin orexin level, which were associated with increased appetite and excess energy intake [38]. In contrast, their physical activity level and energy expenditure were reduced [38], contributing to the development of obesity and DM [38,39].

We found a U-shaped association between nighttime sleep duration and DM risk among Chinese older population, which was consistently demonstrated by several previous literatures [5,11,40]. The increased risk of DM among participants with short nighttime sleep duration might be related to the activation of sympathetic nervous system and increased cortisol level [41], which could inhibit pancreatic function and cause glucose intolerance, and ultimately lead to DM [40,41]. In addition, short nighttime sleep was found to be associated with high circulating levels of IL-6 and tumor necrosis factor-α levels [42,43]. The increased level of inflammation could further impair glucose stability and beta-cell function [37]. Studies also reported that short sleep duration might decrease serum leptin level and increase serum ghrelin level [44,45], which could subsequently cause enlarged adiposity and eventually increase the risk of DM [46,47]. Moreover, people with short sleep duration were more likely to engage in some risky health behaviors, such as lower fruit consumption, irregular eating pattern, less physical activity [48,49], and reduced energy expenditure [50,51], which were all associated with the development of DM [38,52]. In terms of the association between long nighttime sleep duration and increased risk of DM, it might be explained by the increased levels of inflammatory cytokines [53,54]. In addition, evidence showed that people with long nighttime sleep were more likely to have poor sleep quality, such as sleep fragmentation [55], which was related to impaired glycemic control and increased risk of DM [56]. Furthermore, long sleepers were more likely to have symptoms of insomnia and OSA [57], which were well-established risk factors of DM [58]. In addition, they tended to adopt a sedentary lifestyle due to tiredness and frequent sleepiness [59,60], which could also partially contribute to the development of DM. Nevertheless, future studies are warranted to further elucidate the underlying mechanism.

The combined effects of daytime napping and nighttime sleep duration on DM risk among older population in our study was not fully consistent with previous publications [6,12,24]. A study in the US found a dose-response relationship between daytime napping and higher DM risk in each subgroup of nighttime sleep [6]. In contrast, we only find similar trend in participants with nighttime sleep duration ≤4 h or between 6–8 h. We even found that long daytime napping over 1 h per day had a smaller risk estimates compared to non-nappers among participants slept for 4–6 per night. The exact underlying mechanism for the inconsistency is unknown. In our study population, the mean 24-h sleep duration of participants with >1 h daytime napping and 4–6 h nighttime sleep was 7.5 h (SD: 0.6 h), which was comparable to participants in the reference group (mean: 7.6 h, SD: 0.6 h). Therefore, we speculated that daytime napping might in part compensate for less sleep at night, thus leading to an optimal overall sleep duration in a 24-h period and therefore reducing the risk of DM. We further showed that long daytime napping over 1 h and short nighttime sleep duration ≤4 h had the highest risk of DM, which was different from the findings in other two studies [12,24]. It might be explained by the older study population, differences in categories of daytime napping and nighttime sleep duration, as well as different covariates adjusted in the analysis. Future studies are needed to confirm our findings.

Our study had a longitudinal design, which allowed us to explore the potential temporal associations of daytime napping and nighttime sleep duration on the incidence of DM in Chinese elderly. We also used several criteria for DM ascertainment according to the American Diabetes Association guidelines, which enabled us to identify undiagnosed DM cases. Nevertheless, several limitations of this study deserved further discussion. Firstly, a majority of participants who did not meet the inclusion criteria or with missing data were excluded from this analysis, which might cause selection bias. Cautions should be taken when extending our findings to other population. Secondly, duration on daytime napping and nighttime sleep were self-reported by participants, which might be subjective to recall bias and lead to misclassification. However, objective assessment of daytime napping or nighttime sleep duration by actigraphy and polysomnography is difficult to be implemented in large-scale epidemiology studies [61,62]. Thirdly, although we have adjusted for several confounders, we cannot rule out the possibility of residual confounding effect due to lack of information [63,64,65,66,67]. For example, physical activity was not included in the analyses, because more than half (57.9%) of the participants did not have data on this information. Furthermore, the modified International Physical Activity Questionnaire (IPAQ)-short form used in CHARLS adopted categorical choices to collect participant’s time spent on different intensity of physical activity, which could lead to an inaccurate estimate of physical activity levels and energy expenditure. Last but not least, frequency of daytime napping was found to be an independent risk factor associated with DM risk [68,69]. However, such information was not available in the current study. Further studies among older Chinese are needed to confirm the associations. 

## 5. Conclusions

This cohort study found that long daytime napping as well as short and long nighttime sleep were associated with increased risk of developing DM among older adults in China. In addition, we observed that long daytime napping and nighttime sleep duration were jointly associated with DM incidence. Interventions targeting at obtaining an optimal daytime napping and nighttime sleep duration among Chinese elderly might have substantial benefit in minimizing the risk of DM. Our study also supports that daytime napping-related measures should be included in sleep studies.

## Figures and Tables

**Table 1:** Comparison of baseline characteristics according to different daytime napping duration.

Baseline Characteristics	Daytime Napping (h/day)		
	None	≤1	>1
*N*	1234	904	482
**Demographic and lifestyle factors**			
Mean age (years)	66.6 (5.7)	67.1 (5.9)	67.2 (5.8)
Gender, *n* (%) *			
Male	530 (42.9%)	501 (55.4%)	306 (63.5%)
Female	704 (57.1%)	403 (44.6%)	176 (36.5%)
Education, *n* (%) *			
Illiterate/no formal education	778 (63.1%)	487 (53.9%)	248 (51.5%)
Primary school	299 (24.2%)	249 (27.5%)	138 (28.6%)
Middle school or above	156 (12.7%)	168 (18.6%)	96 (19.9%)
Race, *n* (%) *			
Han ethnicity	1101 (92.8%)	841 (96.1%)	437 (94.8%)
Other minorities	85 (7.2%)	34 (3.9%)	24 (5.2%)
Area of residence, *n* (%) *			
Urban	866 (70.2%)	592 (65.5%)	340 (70.5%)
Rural	368 (29.8%)	312 (34.5%)	142 (29.5%)
Current marital status, *n* (%)			
Not married	224 (18.2%)	146 (16.2%)	82 (17.0%)
Married or cohabitated	1010 (81.8%)	758 (83.8%)	400 (83.0%)
Ever smoker, *n* (%) *	489 (39.6%)	398 (44.0%)	255 (52.9%)
Ever drinker, *n* (%) *	484 (39.3%)	420 (46.5%)	253 (52.5%)
Mean sleep duration (hour) *	5.9 (2.1)	6.2 (1.9)	6.7 (1.9)
Sleep duration groups, *n* (%) *			
≤4 h	389 (31.5%)	347 (38.4%)	221 (45.9%)
4–6 h	426 (34.5%)	329 (36.4%)	142 (29.5%)
6–8 h	316 (25.6%)	166 (18.4%)	62 (12.9%)
>8 h	103 (8.3%)	62 (6.9%)	57 (11.8%)
**Clinical/biochemical measures**			
BMI (kg/m^2^) *	22.5 (3.6)	22.9 (3.7)	23.2 (3.6)
Obesity, *n* (%)	79 (7.0%)	75 (9.0%)	38 (8.7%)
Waist circumference (cm) *			
Male	82.9 (9.4)	83.6 (9.3)	85.7 (9.6)
Female	84.3 (9.9)	86.6 (10.5)	86.7 (11.1)
Central obesity, *n* (%)	415 (37.3%)	333 (40.4%)	188 (43.1%)
Systolic BP (mmHg)	133.5 (22.6)	132.4 (21.9)	134.6 (22.4)
Diastolic BP (mmHg)	74.9 (11.9)	73.8 (11.7)	75.0 (11.2)
Plasma glucose (mmol/L)	5.7 (0.8)	5.7 (0.7)	5.7 (0.8)
HbA1c (%) *	5.1 (0.4)	5.1 (0.4)	5.2 (0.4)
Total cholesterol (mmol/L)	5.0 (1.0)	5.0 (1.0)	4.9 (1.0)
Triglycerides (mmol/L)	1.34 (0.8)	1.32 (0.8)	1.34 (0.8)
HDL-cholesterol (mmol/L) *	1.4 (0.4)	1.4 (0.4)	1.3 (0.4)
LDL-cholesterol (mmol/L)	3.1 (0.9)	3.1 (0.9)	3.0 (0.9)
**Outcomes**			
DM, *n* (%)	155 (12.6%)	123 (13.6%)	80 (16.6%)

Abbreviation: BMI: body mass index; BP: blood pressure; HbAlc: hemoglobin A1c; HDL: high-density Lipoprotein; LDL: low-density lipoprotein; and DM: diabetes mellitus. Data were reported as mean (SD) or number (percentage). Missing data: education: *N* = 1; race: *N* = 98; drinking status: *N* = 2; BMI: *N* = 224; waist circumference: *N* = 247; systolic BP: 222; diastolic BP: *N* = 222. * *p* < 0.05 for difference.

**Table 2:** Comparison of baseline characteristics according to different nighttime sleep duration.

Baseline Characteristics	Nighttime Sleep Duration (h/day)			
	≤4	4–6	6–8	>8
*N*	544	897	957	222
**Demographic and lifestyle factors**				
Mean age (years) *	67.4 (5.9)	66.8 (5.9)	66.5 (5.5)	67.7 (6.0)
Gender, *n* (%) *				
Male	228 (41.9%)	469 (52.3%)	521 (54.4%)	119 (53.6%)
Female	316 (58.1%)	428 (47.7%)	436 (45.6%)	103 (46.4%)
Education, *n* (%) *				
Illiterate/no formal education	384 (70.6%)	479 (53.4%)	513 (53.7%)	137 (61.7%)
Primary school	109 (20.0%)	238 (26.5%)	282 (29.5%)	57 (25.7%)
Middle school or above	51 (9.4%)	180 (20.1%)	161 (16.8%)	28 (12.6%)
Race, *n* (%) *				
Han ethnicity	496 (95.2%)	822 (95.1%)	869 (94.0%)	192 (90.1%)
Other minorities	25 (4.8%)	42 (4.9%)	55 (6.0%)	21 (9.9%)
Area of residence, *n* (%) *				
Urban	401 (73.7%)	572 (63.8%)	653 (68.2%)	172 (77.5%)
Rural	143 (26.3%)	325 (36.2%)	304 (31.8%)	50 (22.5%)
Current marital status, *n* (%) *				
Not married	110 (20.2%)	143 (15.9%)	141 (14.7%)	58 (26.1%)
Married or cohabitated	434 (79.8%)	754 (84.1%)	816 (85.3%)	164 (73.9%)
Ever smoker, *n* (%) *	213 (39.2%)	383 (42.7%)	445 (46.5%)	101 (45.5%)
Ever drinker, *n* (%) *	219 (40.3%)	408 (45.5%)	424 (44.4%)	106 (47.7%)
Mean daytime napping (hour) *	0.49 (0.03)	0.63 (0.02)	0.77 (0.03)	0.79 (0.06)
**Clinical/biochemical measures**				
BMI (kg/m^2^)	22.5 (3.7)	22.8 (3.8)	23.0 (3.5)	22.6 (3.6)
Obesity, *n* (%)	36 (7.3%)	68 (8.3%)	73 (8.3%)	15 (7.5%)
Waist circumference (cm)				
Male *	81.9 (9.0)	83.8 (9.7)	84.4 (9.4)	84.5 (9.3)
Female	84.6 (10.1)	85.8 (10.5)	85.7 (10.4)	85.2 (9.9)
Central obesity, *n* (%)	179 (36.2%)	321 (39.8%)	353 (40.5%)	83 (41.1%)
Systolic BP (mmHg)	133.7 (23.4)	133.0 (22.7)	133.1 (21.3)	134.7 (22.6)
Diastolic BP (mmHg)	75.0 (12.3)	74.1 (11.4)	74.4 (11.6)	75.5 (12.4)
Plasma glucose (mmol/L)	5.7 (0.8)	5.7 (0.7)	5.7 (0.8)	5.7 (0.9)
HbA1c (%)	5.1 (0.4)	5.1 (0.4)	5.1 (0.4)	5.1 (0.4)
Total cholesterol (mmol/L)	5.0 (1.0)	5.0 (1.0)	5.0 (1.0)	5.1 (1.0)
Triglycerides (mmol/L)	1.36 (0.9)	1.28 (0.7)	1.37 (0.8)	1.30 (0.8)
HDL-cholesterol (mmol/L) *	1.4 (0.4)	1.4 (0.4)	1.3 (0.4)	1.4 (0.4)
LDL-cholesterol (mmol/L)	3.1 (0.9)	3.1 (0.9)	3.1 (0.9)	3.1 (0.9)
**Outcomes**				
DM, *n* (%)	87 (16.0%)	109 (12.2%)	125 (13.1%)	37 (16.7%)

Abbreviation: BMI: body mass index; BP: blood pressure; HbAlc: hemoglobin A1c; HDL: high-density lipoprotein; LDL: low-density lipoprotein; DM: diabetes mellitus. Data were reported as mean (SD) or number (percentage). * *p* < 0.05 for difference.

**Table 3:** Associations between daytime napping and nighttime sleep duration with the risk of diabetes mellitus.

Independent Variable	Crude Model	Adjusted Model 1 ^†^	Adjusted Model 2 ^§^	Adjusted Model 3 ^ξ^
	RR (95%CI)	RR (95%CI)	RR (95%CI)	RR (95%CI)
**Daytime napping (h/day)**				
None	1 (ref)	1 (ref)	1 (ref)	1 (ref)
≤1	1.10 (0.85, 1.41)	1.09 (0.83, 1.43)	1.12 (0.85, 1.47)	1.07 (0.81, 1.42)
>1	1.39 (1.03, 1.86) *	1.60 (1.17, 2.19) *	1.63 (1.18, 2.24) *	1.52 (1.10, 2.10) *
**Nighttime sleep duration (h/day)**				
≤4	1.27 (0.94, 1.70)	1.28 (0.93, 1.76)	1.35 (0.98, 1.87)	1.45 (1.04, 2.01) *
4–6	0.92 (0.70, 1.21)	0.92 (0.69, 1.24)	0.96 (0.71, 1.29)	0.97 (0.72, 1.31)
6–8	1 (ref)	1 (ref)	1 (ref)	1 (ref)
>8	1.33 (0.89, 1.99)	1.52 (1.00, 2.32) *	1.52 (1.00, 2.31) *	1.55 (1.01, 2.38) *

^†^ Adjusted Model 1: adjusted for age, gender, race, education level, area of residence, marital status, drinking and smoking status, systolic BP. ^§^ Adjusted Model 2: additionally adjusted for nighttime sleep duration when the independent variable was daytime napping, and vice versa. **^ξ^** Adjusted Model 3: additionally adjusted for BMI and total cholesterol level. Abbreviation: RR: relative risk. * *p* < 0.05.

## Data Availability

The data underlying this article are available in a public, open access repository, and can be accessed at China Health and Retirement Longitudinal Study (CHARLS) http://charls.pku.edu.cn/index/en.html (access on 15 September 2020).

## Supplementary Materials

The following are available online at https://www.mdpi.com/article/10.3390/ijerph18095012/s1, Figure S1: Plots of estimated smoothing spline function of nighttime sleep duration with 95% confidence band for the generalized additive model when the response variable was DM, Table S1: STROBE Statement—Checklist of items that should be included in reports of cohort studies, Table S2: Associations of independent variables and DM measured by the multivariable generalized additive model.
