# Supplementary material for: Daytime Napping and Nighttime Sleep Duration with Incident Diabetes Mellitus: A Cohort Study in Chinese Older Adults
Source: Int J Environ Res Public Health. 2021 May 9;18(9):5012. doi: 10.3390/ijerph18095012 (PMC8125963; doi:10.3390/ijerph18095012)
Supplement: Supplementary file 1 [file ijerph-18-05012-s001.zip › Supplementary Figure S1.pdf]

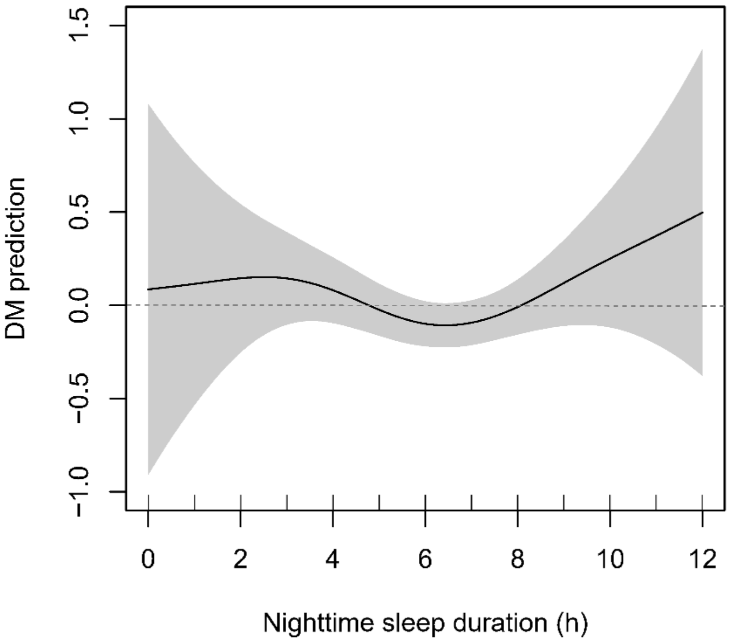

Supplementary Figure S1. Plots of estimated smoothing spline function of nighttime sleep duration with 95% confidence band for the generalized additive model when the response variable was DM.
