# Supplementary material for: Daytime Napping and Nighttime Sleep Duration with Incident Diabetes Mellitus: A Cohort Study in Chinese Older Adults
Source: Int J Environ Res Public Health. 2021 May 9;18(9):5012. doi: 10.3390/ijerph18095012 (PMC8125963; doi:10.3390/ijerph18095012)
Supplement: Supplementary file 1 [file ijerph-18-05012-s001.zip › Supplementary Table S2.pdf]

Supplementary Table S2. Associations of independent variables and DM measured by the multivariable generalized additive model

	Outcome: DM	
	β	***p***
Age	0.006	0.617
Gender		
Male	Ref	
Female	0.420	0.020*
Education		
Illiterate/no formal education	Ref	
Primary school	0.179	0.229
Middle school or above	0.074	0.697
Race		
Han ethnicity	Ref	
Other minorities	-0.046	0.865
Area of residence		
Rural	Ref	
Urban	0.211	0.110
Current marriage status		
Not married	Ref	
Married or cohabitated	-0.186	0.257
Ever smoker	0.060	0.717
Ever drinker	0.019	0.894
SBP	0.004	0.164
Daytime napping	0.003	0.010*
Nighttime sleep duration	Smooth Curve, EDF = 1.951	0.605

Adjusted for age, gender, race, education level, area of residence, marital status, drinking and smoking status and systolic BP. *P <0.05.
